# The Impact of Immune System in Regulating Bone Metastasis Formation by Osteotropic Tumors

**DOI:** 10.1155/2015/143526

**Published:** 2015-05-03

**Authors:** Lucia D'Amico, Ilaria Roato

**Affiliations:** ^1^Department of Orthopedics, Washington University School of Medicine, St. Louis, MO 63130, USA; ^2^CeRMS, San Giovanni Battista General Hospital, University of Turin, Via Santena 5, 10126 Turin, Italy

## Abstract

Bone metastases are frequent and debilitating consequence for many tumors, such as breast, lung, prostate, and kidney cancer. Many studies report the importance of the immune system in the pathogenesis of bone metastasis. Indeed, bone and immune system are strictly linked to each other because bone regulates the hematopoietic stem cells from which all cells of the immune system derive, and many immunoregulatory cytokines influence the fate of bone cells. Furthermore, both cytokines and factors produced by immune and bone cells promote the growth of tumor cells in bone, contributing to supporting the vicious cycle of bone metastasis. This review summarizes the current knowledge on the interactions among bone, immune, and tumor cells aiming to provide an overview of the osteoimmunology field in bone metastasis from solid tumors.

## 1. Introduction

The skeleton is the most common site of metastasis and bone in turn is the main responsible of death since the presence of bone metastases makes the primary disease no longer curable [1]. Symptoms like bone pain, hypercalcemia, fracture, and spinal cord compression appear in this type of metastasis, causing a decline in the quality of life [2]. Some types of tumors are characterized by a selective bone tropism, out of which are prostate, breast, lung, and kidney cancers. Bone metastases can give osteolytic, osteosclerotic, or mixed lesions. Osteolytic metastases are due to an enhanced activity of bone-resorbing cells, the osteoclasts (OCs), which cause bone destruction [3, 4]. Typically, breast, lung, and kidney cancers metastasize to bone with osteolytic lesions, whereas prostate cancer metastasizes with osteosclerotic ones. Lung and kidney tumors metastasize in an early phase of the disease, while breast cancer metastasizes with a slower and less aggressive growth. At least 65–75% of breast and prostate cancer patients develop bone metastases during the course of their disease, and breast cancer patients show a relatively long median survival time after diagnosis of bone metastases [5–7]. Bone metastases from prostate cancer are typically osteosclerotic and are caused by an increased activity of bone-forming cells, the osteoblasts (OBs), leading to enhanced bone formation [3, 4].

Approximately 30–40% of NSCLC patients develop bone metastasis during their disease, with a median survival time measured in months [1]. About 20–35% of kidney cancer patients develop bone metastases, which are particularly destructive, with a rate of skeletal complications higher than other tumors [8, 9]. Accumulating evidences suggest the importance of the immune cell response to factors in the tumor microenvironment as main regulator of cancer progression and metastases. The bone marrow is a reservoir for immune cells such as macrophages, dendritic cells (DCs), myeloid derived suppressor cells (MDSCs), and different T cell subsets that can directly impair the so called “tumor/bone vicious cycle” [10]. This review focuses on the current knowledge of the role of the immune cells in controlling tumor spreading to bone.

## 2. Bone Marrow Is an Attractive Soil for Cancer Cells

Bone marrow (BM) microenvironment is a fertile soil for homing, survival, and proliferation of circulating cancer cells. It provides both endosteal and vascular niches, which support hematopoietic and nonhematopoietic stem cells such as mesenchymal stem cells [11]. OCs degrade endosteal components and promote mobilization of hematopoietic progenitor cells [12], whereas OBs on the endosteal surface of bone are critical supporting cells for hematopoietic stem cells (HSCs) in BM [13, 14]. Indeed, stimulation of the PTH (parathyroid hormone) receptor on OBs increased the number of HSCs in BM [13] and also the size of HSC niche, which promotes skeletal localization of prostate cancer cells [15]. Shiozawa et al. demonstrated that, after injection in a mouse model of bone metastasis, human prostate cancer cells occupy mouse HSC niche, displacing HSCs. Thus, the HSC niche is a direct target of prostate cancer cells during dissemination and plays a pivotal role in bone metastases [16]. BM tissue is constituted by red and yellow marrows. Red marrow contains HSCs and yellow marrow mainly consists of fat cells [17]. Red marrow is particularly vascularised; thus, it is a common site of metastasis. Recently, an important role of yellow marrow in the pathogenesis of bone metastasis has also been recognized because bone marrow adipocytes promote the growth of metastatic tumor cells in bone [18].

**Figure 1:**
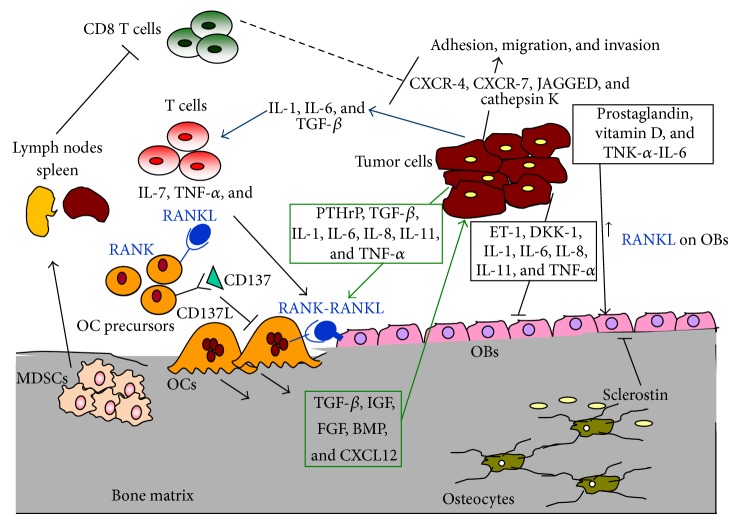
Interactions among bone, immune, and tumor cells sustain the vicious cycle of bone metastasis. Tumor cells release cytokines that activate T cells to produce proosteoclastogenic factors, such as RANKL, which activate OCs. In turn, the release of bone matrix growth factors during bone resorption enhances the tumor growth. MDSCs originate from BM and migrate to secondary lymphoid organs where they inhibit the antitumor immune response mediated by CD8 T cells. Consequently, the increased tumor growth induces the production of osteolytic factors which activates the OCs, the cells responsible for bone destruction.

In physiological conditions, bone undergoes a constant remodelling through OC-mediated bone resorption and OB-mediated bone regeneration in a coupled manner to maintain homeostasis. However, during tumor growth in the bone, dysregulation of this process leads to osteolytic or osteosclerotic phenotypes [19]. Indeed, cancer cells express adhesion molecules which bind their ligand on BM stromal cells, releasing angiogenic and bone-resorbing factors, which disrupt the normal homeostasis of BM microenvironment causing bone metastasis (Figure 1) [20, 21]. For instance, vascular endothelial molecule-1 (VCAM-1) is expressed in breast cancer and binds *α*4*β*7 and *α*4*β*1 (VLA-4) integrins on OC precursors with high affinity, causing osteoclastogenesis. *α*4 or VCAM-1 blocking antibodies effectively inhibit bone metastasis [22]. *αvβ*3 is another integrin expressed by breast cancer cells [23], which is particularly important for OC adhesion to bone [23]. CD44 is a molecule highly expressed by breast cancer cells, which promotes invasion and adhesion to BM [24]. Moreover, CD44 is also expressed by breast cancer stem cells which can lie in a dormant state in the BM [25] and then directly induce bone metastasis [26].

CXCL12, expressed by OBs and endothelial cells in BM, and its receptors CXCR-4 regulate cell migration and bone metastasis from prostate cancer [27, 28]. When cancer cells reach the bone microenvironment, they induce the release of different factors enmeshed in the bone matrix, such as bone morphogenetic proteins (BMPs), transforming growth factor-*β* (TGF-*β*), insulin-like growth factor (IGF), and fibroblast growth factor (FGF) as well as others that stimulate the growth of metastatic tumor cells (Figure 1) [29]. The last, in turn, secretes prostaglandins, PTH, PTH-related peptide, activated vitamin D, interleukin-6 (IL-6), and tumor necrosis factor (TNF), leading to an increase in receptor activator of nuclear factor NF-kB ligand (RANKL) expression on OBs and BM stromal cells [4], which stimulates the OC number and survival and activity (Figure 1). Interestingly, prostate and breast cancer cells respond to these factors activating different OB transcription factors [30, 31]; thus cancer cells can differentiate into an osteoblastic bone-forming phenotype. This phenomenon is called osteomimicry and it has been observed in bone metastatic prostate and breast cancer cell lines [32–34].

Progenitors and mature cells in the BM frequently expressed the receptor Notch [35], whereas the Notch ligand Jagged is overexpressed by bone metastatic tumor cells [36]. Thus, cancer cells directly activate osteolysis through the Notch-Jagged interactions in the BM. In particular, Jagged1, which is a downstream mediator of the prometastatic TGF-*β*, directly activates OC differentiation and promotes tumor growth stimulating IL-6 production by OBs [37].

## 3. Bone and Immune System Cross Talk

Bone and immune and hematopoietic systems are tightly linked since bone cells and hematopoietic cells are in deep physical contact, are reciprocally regulated, are interconnected in their function, and share several common pathways [38]. Indeed, bone cells express surface molecules regulating the expansion of HSCs from which all cells of the mammalian immune system derive, whereas many immunoregulatory cytokines directly act on bone cells [39, 40]. OBs and OCs both affect the maintenance and the mobilization of HSCs [13, 14]. OBs control the proliferation of hematopoietic progenitors [41] and support commitment and differentiation of all stages of B cell development. Indeed* in vitro* production of B cell precursors from progenitors required contact with OBs and expression of CXCL12 and interleukin-7 (IL-7), which was induced by PTH [13, 42]. Moreover, OC precursors, T, B, and NK cells originate from the same stem cell; thus, some of the receptors and ligands that mediate the immune process also regulate the maturation of OC precursors and the ability of OCs to degrade bone. Circulating OC precursors are a reservoir of the pre-OC pool in the BM, but they are also an abundant source of pre-OCs that can be recruited into bone or joint tissue in response to reparative or pathological signals.

RANKL, its receptor RANK, and the natural decoy receptor osteoprotegerin (OPG) [43] form a crucial molecular link between the immune system and bone [44]. The membrane RANKL is expressed by OBs/stromal cells; the soluble RANKL was originally cloned from T cells [45, 46], but it is also expressed by B cells [47], whereas the receptor RANK is expressed by DCs, monocytes, macrophages, and tumor cells [44, 48]. Activated T cells produce RANKL, which directly regulates osteoclastogenesis and bone remodelling, explaining why different pathological conditions, such as cancer, result in systemic and local bone loss. The RANKL to OPG ratio in serum is a determinant factor for OC activation at bone level: a higher serum RANKL to OPG ratio is an index for upregulation of osteoclastogenesis [49].

Many immune factors, including costimulatory receptors and cytokines such as interferon-*γ* (IFN-*γ*) and TNF regulate bone cell development, bone turnover, and pathogenesis of bone diseases [50]. The role of IFN-*γ* in osteoclastogenesis is controversial; indeed, in studies conducted* in vitro* [51] and* in vivo*, in animal model, it shows an antiosteoclastogenic effect [52], whereas, in humans, IFN-*γ* increases in presence of bone loss due to oestrogen deficiency and rheumatoid arthritis [53, 54]. IFN-*γ* influences osteoclastogenesis directly by blocking OC formation through inhibition of OC maturation [55] and indirectly by stimulating T cell activation with a consequent increase of proosteoclastogenic factors [56].

### 3.1. The Interactions between T Cells and Osteoclast Precursors Regulate Bone Resorption in Bone Metastasis

A direct regulation of bone resorption by T cell has been widely described for bone metastasis by both solid tumors and multiple myeloma (MM) [57, 58]. Indeed, studies on peripheral blood mononuclear cells (PBMCs), isolated from patients affected by breast, prostate, and lung cancer with bone metastases, demonstrated an increase of circulating OC precursors in these patients compared to patients without bone metastases and healthy controls [58, 59]. In bone metastatic patients, OC precursors differentiate into mature OCs* in vitro* in presence of T cells without adding M-CSF and RANKL, but T cell depletion results in the absence of OC formation without exogenous stimulation [58].

Another important mediator of the interactions between T and bone cells is IL-7, a cytokine produced by stromal cells and by cells at inflammatory site, with different effects on hematopoietic and immunologic systems [60]. The main function of IL-7 is the control of B and T lymphopoiesis [61], but it is also important for the tumor process [62] and the correct bone homeostasis [63, 64]. According to the model considered, IL-7 displays either inhibitory or activator effects on OCs [63, 65]. Some studies demonstrated that IL-7, produced by T cells, promotes osteoclastogenesis by upregulating T cell-derived cytokines, such as RANKL and TNF*α* [66–68], and that its production is increased by oestrogen deficiency [69]. Furthermore, in bone metastatic patients, IL-7 serum levels were significantly higher than those in nonbone metastatic patients and in healthy controls [59, 68, 70]. This increase of serum IL-7 is at least in part dependent on IL-7 production by tumor cells as demonstrated in a human-in-mice model of bone metastasis from lung cancer [71]. All these data confirm the T cell modulatory activity on OCs. Nevertheless, also OCs affect T cell activity, because they present antigenic peptides to T cells and induce FoxP3 expression in CD8 T cells, which regulate an inappropriate activation of the immune response [72]. The cellular responses in cell-to-cell interactions between T cells and OCs are regulated through reciprocal CD137/CD137L and RANK/RANKL interactions [73]. CD137 is a costimulatory member of the TNF receptor induced by T cell receptor activation. Its ligand CD137L is expressed on OC precursors:* in vitro* CD137L ligation suppresses osteoclastogenesis through the inhibition of OCs precursor fusion. On the other hand, RANKL expressed on T cells binds to RANK on OCs, producing a reverse signal in T cells able to enhance apoptosis [73].

### 3.2. T Cells Regulate Tumor Growth in Bone

Many data suggest that T cells can regulate tumor growth in bone also independently from their interaction with bone cells. Indeed, memory T cells have been found in the BM of breast cancer patients suggesting their role in cancer immune surveillance [74]. Moreover, the RANKL-RANK interaction between CD4 T cells and breast cancer cells promotes invasion, dissemination, and metastasis formation in an animal model [75]. Some antibone metastatic therapies show immunomodulatory effects; for instance, the blockade of TGF-*β* at metastatic sites may locally activate an antitumor T cell response, because, normally, TGF-*β*, released in BM by OC activity, inhibits T cell proliferation [76].

Zoledronic acid, an antiresorptive agent, can activate cytotoxic *γ*/*δ*-T cells and inhibit populations of myeloid derived cells with T cell suppressor capabilities [77]. Modulation of antitumor T cell responses alters tumor growth in bone. Indeed, by using mice models Lyn^−/−^, which have more OCs and a hyperactive myeloid population with an increased T cell responses, Zhang et al. reported a reduced tumor growth in bone despite enhanced osteolysis [78]. Lyn is a member of the Src family tyrosine kinases, which inhibits OC differentiation by downregulating PLC*γ*2 activation, which regulates the OC formation and function [79]. PLC*γ*2^−/−^ mice have an increased bone tumor burden despite protection from bone loss, because they have dysfunctional OCs and impaired T cell activation mediated by DCs. Importantly, injection of antigen-specific wild-type cytotoxic CD8 T cells in both these mice models reduces the growth of tumor cells in the bone, regardless of OC functionality. According to these data, a condition of immune deficiency can interfere with the antitumor effects of OC blockade [78]. In particular, cytotoxic CD8 T cells seem to be critical regulators of tumor growth in bone, since their activation diminishes and their depletion enhances bone metastases, even with zoledronic acid.

### 3.3. Myeloid Derived Suppressor Cells Regulate Cancer Progression

Myeloid derived suppressor cells are a heterogeneous population of immature myeloid cells identified by the coexpression of Gr-1 and CD11b in mice and CD11b and CD33 in humans [80–83]. MDSCs are significantly overproduced in tumor-bearing mice and cancer patients and they represent a prognostic indicator in various osteotropic tumors including breast, lung and MM [84, 85]. Emerging evidences suggest the importance of the MDSCs in driving the progression of cancer disease by suppressing both the innate and the adaptive immune response. Thus, MDSCs exert their proneoplastic effects through the impairment of T cell/antigen recognition, the release of small soluble oxidizers, and depletion of essential amino acids from the local extracellular environment [86–88]. Besides suppressing CD4 and CD8 T cell populations, MDSCs promote the activation and expansion of regulatory T cells (Treg) and thus mediate immunosuppression.

Finally, all these mechanisms contribute to tumor progression and metastasis spreading to many organs, especially to bone (Figure 1).

Bone metastases are associated with an increase in OC activation and since MDSCs are progenitors of the OC precursors, it is not surprising that they are found to be largely increased in bone metastatic patients. Strikingly, Sawant et al. confirmed that MDSCs isolated from tumor-bone microenvironment can differentiate into mature and functional OCs* in vitro* and* in vivo* in a mouse model of breast cancer bone metastases [89]. MDSCs from mice bearing bone metastases also induce osteolysis in syngenic animals, indicating that these cells are primed as OC progenitors and the bone microenvironment triggers their activation in functional OCs. It has been also suggested that cancer cells release different soluble factors in the bone, which promotes MDSCs to differentiate into OCs. Thus, breast cancer cells can secrete CCL2, CCL5, or osteopontin which promotes the expression of cathepsin K and matrix metalloproteinase 9 (MMP9), thus enhancing OC functions [90]. On the other hand, MDSC expresses several proosteoclastogenic factors as CCR2, the receptor of CCL2, showing the responsiveness of these cells to the chemokine. Similarly, in the MM model, Zhuang et al. discovered that tumor induced MDSCs were responsible to induce osteolytic lesions by acting as OC precursors [91]. Additionally, only MDSCs isolated from bone are capable of becoming active OCs, suggesting the importance of the bone microenvironment in driving OC maturation.

Despite the critical role for OCs in the establishment of bone metastatic vicious cycle, the PLC*γ*2^−/−^ mouse model, bearing severe OC defects, suggests that MDSCs can enhance tumor growth in bone independently of their ability to differentiate into OCs [78]. Interestingly, the increased PLC*γ*2^−/−^ tumor growth was the result of a higher MDSCs accumulation in secondary lymphoid organs, leading to a strong inhibition of the antitumor T cell response (Figure 1). Despite the importance of MDSCs expansion as a crucial event in the pathogenesis of tumor progression, little is known about the mechanisms leading to this process. Capietto et al. have recently shown *β*-catenin as a crucial modulator of MDSC accumulation in response to tumor [92]. The downregulation of *β*-catenin signaling in MDSC promotes their expansion and consequently increases tumor growth in both mice and humans. On the contrary, expression of a constitutively activate form of *β*-catenin in mice decreased the number of MDSCs and tumor growth. Importantly, the downregulation of *β*-catenin can also occur in MDSCs from WT mice during tumor dissemination to bone, indicating that *β*-catenin pathway modulates MDSC expansion in both primary and metastatic solid tumors.

## 4. Conclusions

The rapidly developing field of osteoimmunology shows the importance of the deep interconnection between skeletal and immune system. This relationship results in the generation of several cellular pathways, which provides the discovery of new potential targets for the prevention and treatment of bone metastasis.

The bone marrow represents an active and hospitable microenvironment, allowing multiple cell interactions which are critical in the pathogenesis of tumor progression. Thus, additional studies to elucidate new mechanisms promoting the accumulation of bone marrow derived cells such as MDSCs are mandatory to address the critical steps of tumor progression in bone. The design of new drugs must consider the potential effects on both immune system and bone; thus further investigations to understand the osteoimmune system are even more important.
